# Relationship between blood clots and COVID-19 vaccines: A literature review

**DOI:** 10.1515/biol-2022-0035

**Published:** 2022-04-26

**Authors:** Seyed Mohammad Hassan Atyabi, Foad Rommasi, Mohammad Hossein Ramezani, Mohammad Fazel Ghane Ezabadi, Mehdi AghaAmooi Arani, Mohammad Hossein Sadeghi, Mohammad Mehdi Ahmed, Amir Rajabi, Nima Dehghan, Ali Sohrabi, Mojtaba Seifi, Mohammad Javad Nasiri

**Affiliations:** School of Medicine, Shahid Beheshti University of Medical Sciences, Tehran, Iran; Faculty of Life Sciences and Biotechnology, Shahid Beheshti University, Tehran, Iran; Microbiology Research Center, Pasteur Institute of Iran, Tehran, Iran; Department of Microbiology, School of Medicine, Shahid Beheshti University of Medical Sciences, Tehran, Iran

**Keywords:** COVID-19 vaccines, thrombotic thrombocytopenia syndrome, blood clots, anti-PF4 antibody, immune system overactivation

## Abstract

SARS-CoV-2 pandemic is one of the most critical pandemics during human civilization. Several therapeutic strategies for COVID-19 management have been offered; nonetheless, none of them seems to be sufficiently beneficial. In effect, vaccines have been proffered as a viable option. The critical issue now is to concentrate on protecting individuals against illness through immunization. One of the causes for concern among the researchers, physicians, and generally the whole community from the onset of vaccination has been the adverse effects (specifically blood clots) that may be observed after the injection of the COVID-19 vaccine. In some countries, such concerns have even resulted in the temporary or permanent discontinuation or abandonment of the application of some vaccines (especially AstraZeneca and Janssen). By evaluating rigorous studies published on this subject, the present article is aimed at identifying the association between blood clot incidence and COVID-19 vaccination. Various methods for producing the COVID-19 vaccines are analyzed, along with their possible pros and cons as well as common and rare side effects, especially VITT and blood clots. Finally, the differences of various vaccines on thrombotic events, WHO recommendations for VITT treatment, and blood clots statics are discussed.

## 1 Introduction

Coronaviruses are numerous enveloped viruses, and their genetic material is made up of ss-RNA [1]. In December 2019, SARS-CoV-2 was discovered following the identification of strange pneumonia in a group of patients in Wuhan, China, Mainland. 2019-novel Coronavirus (nowadays known as SARS-CoV-2) is a human beta-coronavirus responsible for COVID-19, the most sustainable pandemic catastrophe in the last century [2,3]. Apart from the commonly known respiratory symptoms, COVID-19 could cause cardiovascular, neurological, gastrointestinal, and renal complications and involvements, among others. The majority of individuals infected by SARS-CoV-2 are asymptomatic or have mild symptoms. Almost 20% of COVID-19 patients require hospitalization, and nearly 5% could get into critical condition [2]. In the first 6 months of the pandemic, the novel Coronavirus brought about the demise of about one million people and wreaked havoc on the global economy and social order [4]. Since then, several mutations have occurred in the SARS-CoV-2 genome causing the incidence of other variants. Other types of COVID-19 – which originated in the United Kingdom, South Africa, and Brazil, respectively – are more transmissible and virulent, inclusive of B.1.1.7, B.1.351, and P.1. The existence of Spike (S) protein is a defining feature of all coronaviruses, which are linked to severe acute respiratory syndrome (SARS) [5]. The Coronaviruses’ Spike protein assists virus entry through host cells [6,7]. With the development of the pandemic, new variants of SARS-CoV-2 have emerged with mutations in the Spike protein, posing many challenges to the disease management [7,8].

## 2 COVID-19 treatment and management

In the early days of the COVID-19 pandemic, some antibacterial drugs (e.g., teicoplanin and azithromycin) as well as some systemic corticosteroids (e.g., methylprednisolone) were used to control the disease, but were later limited due to the ineffectiveness or widespread side effects [9,10]. Currently, the most common treatments to impede the disease include antiplatelet agents such as aspirin and antiviral drugs such as remdesivir, ribavirin, lopinavir/ritonavir, and umifenovir [11]. Early administration of nitazoxanide has been reported to reduce the viral load in individuals with COVID-19 [12,13]. Nevertheless, there is some controversy over chloroquine and hydroxychloroquine; it has not been particularly effective in recent clinical trials [14,15,16]. Remdesivir is a drug that is now used in many countries. Remarkable recovery from pneumonia and the decreased mortality rate have been observed in the clinical trials following the administration of remdesivir [17,18]. Initially, it was held that ribavirin and lopinavir/ritonavir are able to accelerate the COVID-19 recovery process and reduce the viral load. However, recent clinical studies have detected no effective performance, nor any significant improvement for ribavirin and lopinavir/ritonavir [18,19,20]. Umifenovir has been demonstrated to significantly contribute to clinical improvements such as chest imaging and oxygen saturation [21]. Convalescent plasma (CP) therapy has also been reported to diminish the mortality rate and improve clinical scales. It should be mentioned, however, that further studies have unveiled that the reliability of the evidence on the effect of convalescent plasma in COVID-19 is drastically low and indeterminate [22,23].

Overall, no conclusive and definitive cure has yet been discovered for SARS-COV-2. Therefore, the development of practical medicine and vaccines for the treatment and control of SARS-COV-2 is a priority.

## 3 Vaccination pros

Based on the information obtained from the Centers for Disease Control and Prevention (CDC), approved COVID-19 vaccines will have several benefits for preventing COVID-19. Vaccination aims to suppress the prevalence of COVID-19. In general, it may also protect uninfected individuals against infected ones by interrupting the chain of transmission; therefore, it will be quintessential for hampering the pandemic [8]. Vaccination is a more secure method of boosting immunity and is able to protect people by triggering an antibody (immune system) response without causing illness [24].

## 4 Efficacies of COVID-19 vaccines

The efficacy and impact of the vaccine on the SARS-CoV-2 pandemic are complex, and numerous possibilities could occur following the deployment. Since hospitalization and critical-care admissions put a tremendous burden on the healthcare organizations, the protection capability of a vaccine upon severe disease and mortality is the most substantial endpoint. The promising effect of vaccines on the population can be observed if the vaccination is sufficiently effective in older adults (e.g., >60 years). Vaccination now is widely distributed, including for people who are most susceptible to COVID-19. Admittedly, vaccines do not improve the clinical course but relieve the SARS-CoV-2 transmissibility [4].

## 5 Various types of COVID-19 vaccine

A very brief overview of the potential primary vaccines will be provided with a focus on the vaccines’ safety and influence records in the following sections.

### 5.1 mRNA-based vaccines

The mechanism behind the mRNA vaccination is that mRNA is an intermediary messenger that must be converted into an antigen after being delivered into host cells via multiple pathways. Pfizer and Moderna, as potential candidates for COVID-19 mRNA vaccines, exert their effect via injecting SARS-CoV-2 spike protein-encoding mRNA directly into the host cell. Although pure mRNA has been swiftly degraded over the last decade, various technical progressions in transfer systems and RNA carriers have enabled fast and secure uptake of mRNA into the cytosol, where ribosomes transform mRNA into a protein that could trigger an immunological reaction [25]. Theoretically, this method has a variety of advantages over the more traditional vaccination types. First, its ease and high speed with which it can trigger protein synthesis in the host cell can be counted. Second, immediately after the transfection, the target protein is expressed through translation from mRNA. Finally, protein-based vaccinations are frequently made by bacteria. In contrast, mRNA vaccines are typically translated by the host translation machinery, leading to an antigen that closely resembles the protein structure generated from the viral genome (e.g., posttranslational modifications) [26,27].

### 5.2 Modified Adenovirus vector-based vaccines

The global requirement for mass immunization against the COVID-19 pandemic is the main impetus for developing adenovirus vector vaccines [28]. Phase III of clinical trial studies has been completed for several vaccines based on the full-length S protein and nonreplicating adenovirus vectors. These vaccines include sputnik V, AstraZeneca (ChAdOx1-S/AZD1222), CanSino, and Janssen (Ad26.COV2) [29].

Different adenoviruses are utilized as vectors in modern COVID-19 vaccines for the mentioned vaccines; nonetheless, the primary manufacturing platforms and mechanism of action are the same in all of them. For example, Janssen is based on a human adenovirus, whereas Oxford vaccination is based on a chimpanzee (ChAdOx1) adenovirus. The Oxford approach used a chimpanzee virus to decrease the impact of human adenovirus antibodies over time. The SARS-CoV2 Spike protein gene is produced as DNA; then, the synthesized DNA is inserted into the DNA genome of adenoviruses, substituting a crucial adenovirus gene (E1) for virus replication; adenovirus cannot multiply. After this change, it cannot initiate a complete infection cycle as a result of this alteration. However, it may penetrate cells and show the inserted unfamiliar gene to make the S protein of Coronavirus [29]. High dosages of vector particles are required for effective adenovirus vector vaccinations. When these particles penetrate host cells, they are identified by innate immunity receptors, causing the production of cytokines and chemokines responsible for the adverse effects of vaccination [28,30].

### 5.3 Subunit vaccines

The organism’s purified protein extracts – usually administered together with an adjuvant to elevate the immune response – are a more traditional method of advancing in vaccines. So far, only NVX-CoV2373 has passed the third phase of the clinical trials [31]. Novavax vaccine has a recombinant full-length S protein as an antigen generated in Sf9 insect cells with stabilizing mutations. Adenovirus vector and inactivated vaccines preserve the vaccine at 4°C for extended cycles due to its durability. It has huge advantages over similar approved mRNA vaccines that need low freezing temperatures for storage [32].

### 5.4 Live-attenuated and inactivated virus vaccines

**Figure 1:**
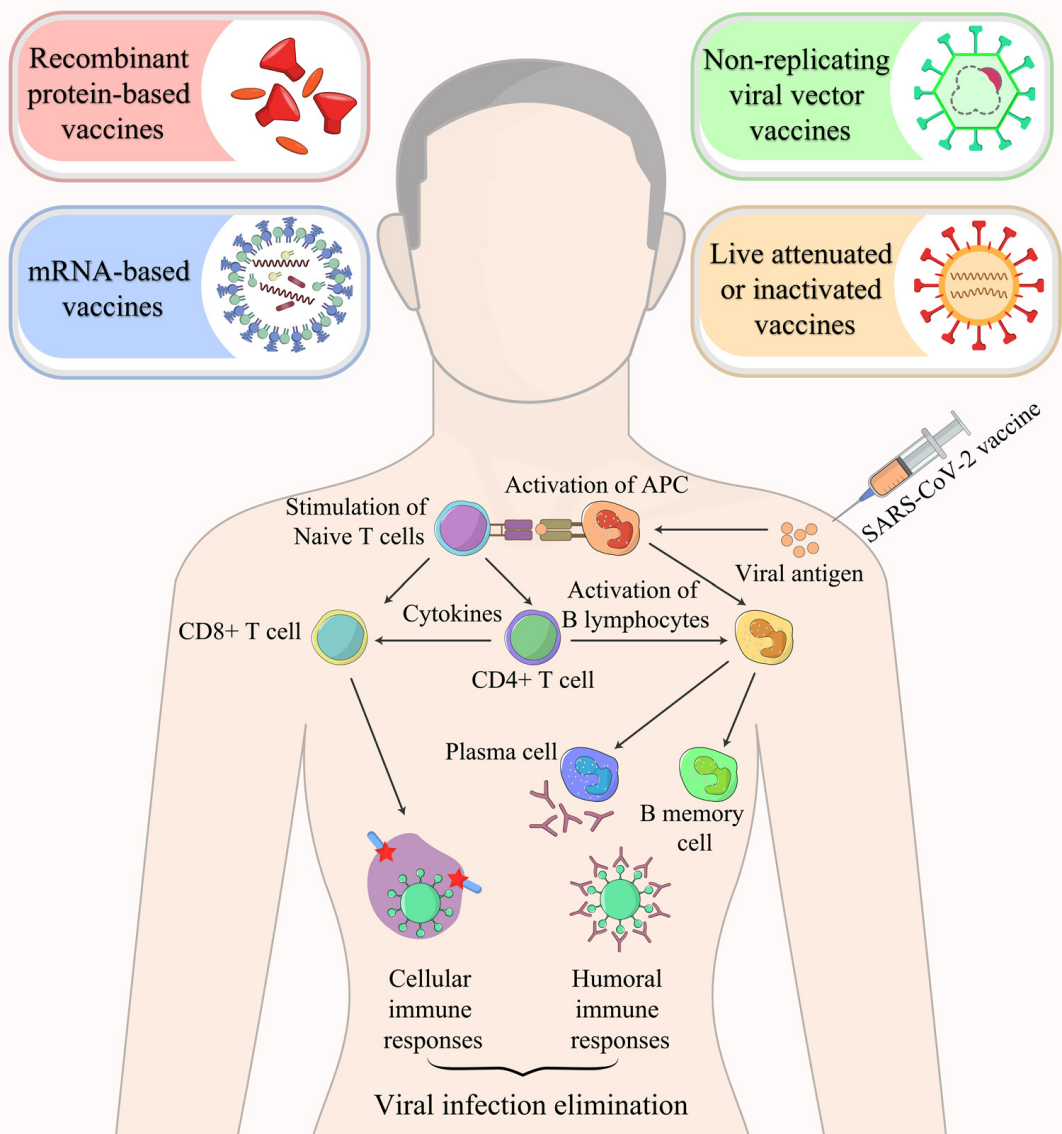
COVID-19 vaccines can be synthesized by employing various methods. All SARS-CoV-2 vaccines aim to present a viral antigen to the immune system, resulting in cellular and humoral immune responses. The process of cellular and humoral immune responses activation is illustrated in detail.

The available inactivated vaccines made by China, the Indian vaccine named Bharat Biotech (Bharat, Hyderabad, Telangana, India), and Valneva vaccine in development by the European Union all are created using very comparable and well-established technology. Generated in Vero cells, the virus is chemically inactive and is purified to varying degrees, with adjuvants added. In all situations, beta-propiolactone (BPL) is used to attenuate the virus [33]. New research has demonstrated that the BPL-inactivated molecular configuration of SARS-CoV2 could be cause for concern since it is found that almost all spikes had acquired a post-fusion shape [34]. Different methods that are adopted to produce COVID-19 vaccines are shown in Figure 1.

## 6 Side effects of COVID-19 vaccines

Some side effects have been reported after receiving COVID-19 vaccines, most of which are short term and resolve after a few days without any medical intervention. However, some more severe side effects have also been reported.

The findings by the Brighton Collaboration as well as other previous studies on vaccines’ side effects are briefly mentioned here: various types of strokes like nonhemorrhagic and hemorrhagic, encephalomyelitis, appendicitis, immune thrombocytopenia, Bell’s palsy, acute myocardial infarction, anaphylaxis, deep vein thrombosis, pulmonary, myocarditis or pericarditis, disseminated intravascular coagulation, and narcolepsy [35].

## 7 Short-term and common side effects

Common side effects after receiving the first dosage of COVID-19 vaccine include about 63.7% injection site tenderness, 54.2% injection site pain, 53.1% fatigue, nearly 52.6% headache, 44.2% malaise, 44.0% myalgia, about 33.6% pyrexia and feverishness, and 7.9% fever >38 °C [36].

Flu-like symptoms such as joint and muscle pain or headache are common side effects and could last for 1–2 days after vaccination [37]. In South Africa, a possibly vaccine-related severe adverse effect was confirmed 2 days after vaccination of a person who had a fever of more than 40°C but recovered quickly without needing to be hospitalized [38]. According to preliminary evidence from clinical trials in Russia, the most common side effects of the adenovirus-based Sputnik V vaccine are flu-like symptoms and injection-site reactions [39]. Reactions were identified more often after receiving the second dose of the Pfizer-BioNTech vaccine than after receiving the first one [8].

Vaccination has several side effects such as sudden pain in the chest, abdomen, headache, and dizziness. If side effects continue or recur after 3 days, further medical diagnostics should be performed to rule out thrombosis [37].

## 8 Rare severe and long-term side effects

In a clinical trial study in the United Kingdom and Brazil, only 168 participants (among 11,636) experienced severe adverse effects. Seventy-nine participants who had serious side effects had received ChAdOx1 of-19, and 89 of them had received MenACWY [38].

### 8.1 Some of the serious and long-term side effects that have been identified

#### 8.1.1 Anaphylactic shock

There have been reports of allergic reactions including anaphylaxis. The most common adverse reactions to vaccination are local reactions and mild symptoms. Recently, 4.5 per million reports of anaphylaxis have been identified after the administration of both doses [8].

#### 8.1.2 Neurological complications

Neurological symptoms that emerge after the vaccine administration include headache, myalgia, dizziness, muscle spasm, and paresthesia. Sporadic symptoms such as tremors, diplopia, tinnitus, dysphonia, epilepsy, and reactivation of herpes zoster appear after vaccination [8].

A case of transverse myelitis was identified 14 days after receiving the ChAdOx1 nCoV-19 booster vaccine, which is thought to have occurred potentially due to vaccination [38]. Two additional cases of transverse myelitis were initially identified to have been possibly resulted due to vaccination; however, this possibility was later questioned by an independent group of neurological researchers [38]. Researchers have stated that some slight risk of severe neurological disorders (e.g., Bell’s palsy, Guillain‐Barré syndrome (GBS), transverse myelitis, and acute disseminated encephalomyelitis) is caused by vaccination. However, no conclusive clue has been found to link the latter disorders to the vaccine [8].

#### 8.1.3 Problems with blood clotting disorders

Due to the incidence of some instances of DIC and CVT, on March 15, 2021, the European Union put out a motion to temporarily cease the vaccination by AstraZeneca as a precaution, pending the EMA verdict [25]. According to a report, on March 16, about 20 million people in the United Kingdom and European Economic Area had received ChAdOx1 nCoV-19. On the basis of EMA reports, only seven patients had blood clots in multiple vessels (diffuse intravascular coagulation, or DIC), and 18 patients had CVST (cerebral venous sinus thrombosis). While a causal connection to the vaccine has not been established, it is plausible and warrants further investigation [40]. AstraZeneca creates thrombocytopenia and thrombosis in rare cases, such as the cerebral venous sinus or thrombosis in the portal, splanchnic, or hepatic veins. In healthy people, these symptoms appear 5–24 days after the first injection. Most of the patients in that report were women younger than 50 years, and some were prescribed estrogen replacement therapy or oral contraceptives [41].

The specialists on the Committee examined the reports of nine deaths with DIC and CVST recorded by the Member States, most of them being younger than 55 years and women [40]. Recently, the frequency of VITT has been estimated to be about 1 case per 100,000 exposures. However, it is very improbable that several cases of thrombosis will result directly after vaccination, but it should be discovered in a general population year [41].

#### 8.1.4 Prevention and general management of vaccines complications

Factually, the number of confirmed deaths after vaccination is very limited. The majority of them are caused by other reasons (such as underlying illness, accidents, and so on) rather than the vaccine [8,38]. Patients with severe allergies or immediate reaction during 4 hours to polyethylene glycol (PEG) and its derivatives (e.g., poly-sorbates) should not be vaccinated with Pfizer-BioNTech or Moderna mRNA vaccines according to the CDC [8].

Chest or back pain, breathlessness, shoulder swelling or coldness, blurred vision, extreme and deteriorating headache following vaccination, intermittent bleeding, several minor bruising, red or purple stains, and under skin blood blisters are all signs that may signify a clot caused by the COVID-19 vaccine, according to EMA [25]. Anaphylaxis is a fatal side effect, and the vaccinated individuals must be closely monitored and attended to as soon as possible [8].

## 9 Mechanism of thrombotic thrombocytopenia after COVID-19 vaccination

In this section, we first look at a syndrome that clinically resembles postvaccination thrombotic thrombocytopenia. Then, we will review the WHO guideline for the treatment of VITT, and in the end, we will discuss the blood clots statics.

### 9.1 Autoimmune heparin-induced thrombocytopenia

HIT is characterized by unusually severe thrombotic events accompanied by thrombocytopenia. Thrombocytopenia is caused by rogue antibodies directed against platelet factor 4 (PF4), which causes massive platelet aggregation and thrombosis and a reduction in the platelet count resulting in bleeding. As a consequence, patients may experience both severe thrombosis and severe bleeding [42].

In a molecular view, platelet-activating antibodies’ transitory generation of the IgG class – which detects the multimolecular set of the cationic platelet factor 9 (PF4) coupled with poly-anionic heparin – causes heparin-induced thrombocytopenia (HIT), a pro-thrombotic adverse medication event [43].

Heparin-induced thrombocytopenia (HIT) is a pro-thrombotic adverse medication event caused by the transient formation of platelet-activating antibodies from the IgG class, which recognizes multimolecular complexes of (cationic) platelet factor 9 (PF4) coupled with (poly-anionic) heparin. Heparin administration can result in the formation of HIT antibodies since heparin can act as a hapten, and hence, it is recognized by the immune system [44,45]. Platelet factor 4 is heparin bound to a protein, and the immune system produces antibodies against it, causing HIT. These antibodies are mainly of IgG type, and they take around 5 days to form. However, in the last few months, those exposed to heparin may have IgG circulating, as IgG-type antibodies are still generated even after removing their precipitant. In the circulation, IgG antibodies combine with heparin and PF4 to create a complex. The molecular tail of the antibody subsequently attaches to the FcIIA receptor on the platelet’s area of protein. This results in platelet activation, and the formation of platelet micro-particles imports the establishment of blood clots. As a result, the platelet number decreases dramatically, leading to thrombocytopenia. Furthermore, the reticuloendothelial system (mainly the spleen) eliminates antibody-coated platelets and then conducts thrombocytopenia [44,45].

Apart from heparin, other poly-anions (e.g., DNA and RNA, chondroitin sulfate, hyper sulfated, polyphosphates, and bacterial cell wall components) can cause adaptive changes in PF4 required to reveal the HIT antigen. Furthermore, in the absence of additional poly-anions, significant PF4 binding to platelets may result in HIT antigen(s) exposure. In the latter case, poly-anions on the platelet area are likely to increase the proximity of PF4. This issue demonstrates that antigens could be impacted by factors other than heparin administration called autoimmune HIT (A-HIT). These factors could be bacterial wall ingredients or trauma-induced nucleic acid unleash (i.e., a potential cause of spontaneous HIT syndrome), platelet-derived poly-anions, or the AstraZeneca COVID-19 vaccination [43].

### 9.2 Vaccine-induced thrombotic thrombocytopenia

Occasional cases of thrombotic thrombocytopenia caused by the resistance of platelet-activating antibodies against PF4 may happen after inoculation with ChAdOx1 n-Cov-19. The clinical sight of moderate-to-severe thrombocytopenia and thrombotic consequences at uncommon locations happening 1–2 weeks after immunization with ChAdOx1 n-Cov-19 against SARS-CoV-2 indicates a syndrome that clinically mimics severe heparin-induced thrombocytopenia. According to one study, five patients infected by venous thrombosis and thrombocytopenia 7–10 days after injecting the first AstraZeneca dose of adenoviral vector vaccine showed significant levels of antibodies to platelet factor 4-poly-anion complexes, without any prior heparin exposure. Antibodies to SARS-CoV-2 nucleocapsid protein were negative in all five individuals. Thus, the previous infection with SARS-CoV-2 could be deemed to have been highly unlikely. In a population of about 130,000 vaccinated individuals, only five cases of VITT were observed. The clinical parameters compared to those of immune system heparin-induced thrombocytopenia were distinguished within the vaccine-induced resistant thrombotic thrombocytopenia patients. Scientists conjecture that these complications are vaccine-induced thrombotic thrombocytopenia (VITT), an infrequent vaccine-related form of spontaneous heparin-induced thrombocytopenia [46].

## 10 Mechanism of VITT

The pathogenic mechanism of VITT, which the German Greifswald Company recently explained by Andreas Greinacher, is similar to heparin-induced thrombocytopenia (HIT). In this complication, IgG-type antibodies cause a pro-thrombotic condition that detects multimolecular complexes between the cationic platelet factor 4 and the anionic heparin and trigger platelet activation via the FcRIIA receptor. However, this syndrome is unusual since it occurs in immunized people with COVID-19 who have not received any heparin therapy during their lives. For the formation of this prothrombotic condition, known as “spontaneous” or autoimmune HIT, some explanations can be provided. It has recently been discovered that factors except heparin can produce a progressive syndrome with clinical and laboratory characteristics that closely mimic HIT. The mentioned factors include certain highly sulfated and highly negatively charged oligosaccharides (e.g., Pentosan poly-sulfate, hyper-sulfated chondroitin sulfate, and other similar compounds) [46]. A HIT-like phenomenon has been documented after knee substitution surgery and bacterial or viral infections in addition to poly-anionic drug exposure. These results are consistent with PF4’s antibacterial and anticoagulant potential. Indeed, active platelets unleash PF4 in response to pathogens, which aids neutrophil recruitment and enables neutrophil exocytosis to unleash myeloperoxidase and lysozyme.

Furthermore, PF4 binds directly to bacteria, forming a neo-antigen recognized by anti-PF4/heparin or anti-PF4/poly-anion antibodies. This will result in immune complexes that are important in antibacterial host defense. Many scientists have been researching COVID-19, and they have found nonplatelet-activating and platelet-activating anti-PF4/heparin antibodies. The aforementioned mechanisms of two antibodies could also be implicated in the antiviral response to SARS-CoV-2 infection [47].

## 11 Anti-PF4 antibody inducers

The inflammatory stimulation of the immunization or vaccination activates anti-PF4 antibodies, which cross-reacts with PF4 and platelets in the cases of VITT syndrome. It has been discovered that AstraZeneca COVID-19 vaccination increased the reactivity of VITT patients’ serum with platelets, implying the interactions between the vaccine and platelets or the vaccine and PF4 [43].

A theory could be buttressed by adenovirus utilized as a vector in AstraZeneca vaccine: the affinity of adenovirus for PF4 is high and can activate platelets. Free DNA in the vaccination might be a cause of these PF4-reactive antibodies. Previously, it has been demonstrated that DNA and RNA create multimolecular PF4 complexities that fasten antibodies against heparin-induced thrombocytopenia and induce antibodies. Based on what is mentioned, other adenovector vaccines such as Janssen (J&J) and Sputnik V may be potent for causing VITT. Other reports have indicated a similar mechanism; the records state that there may be a link between adenovector vaccines and blood clots [7].

**Figure 2:**
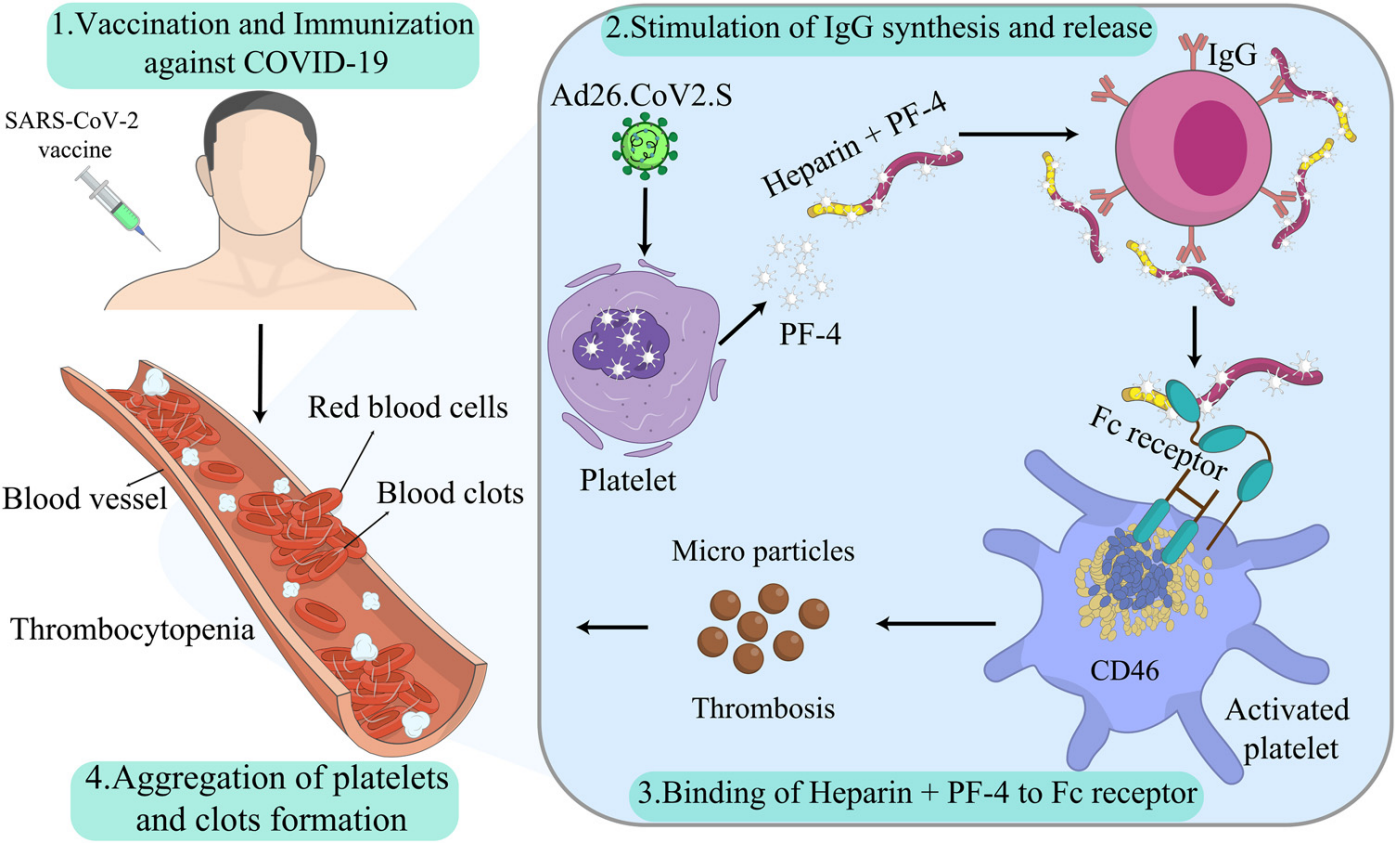
COVID-19 vaccines may cause blood clots: Although VITT is one of the most uncommon side effects of COVID-19 vaccines, it is essential to know the possible mechanisms in which they cause blood clots.

There might be an essential connection between the SARS-COV2 Spike protein itself or similar molecules, with side effects in the body (e.g., thrombosis). Soluble Spike protein has been shown to negatively affect endothelial cells, such as a severe inflammatory response. Furthermore, there are numerous viruses with Spike surface protein in the bloodstream. In practice, all cases of critical SARS-CoV-2 infections (COVID-19) may be exposed to the death danger after thromboembolic events. Even pseudoviruses with Spike protein on their face produce severe inflammatory responses in the tissues and endothelial cells, showing the risk of this protein when it is present systemically. Therefore, different formulations and posttranscription modifications (e.g., capping, splicing, and poly-adenylation) may cause similar heparin–PF4 complex and trigger immune response [7]. The possible mechanism in which COVID-19 vaccines, especially the AstraZeneca vaccine, may cause VITT and blood clots is displayed in Figure 2.

## 12 VITT risk factors

According to several cases, young women who use hormonal contraceptives are more likely to experience harm and adverse reactions to the vaccines [47]. It can be conjectured that the relatively more robust immune systems in women make their bodies more irritable and sensitive; as a result of being more sensitive, the immune stimulant (vaccine) can cause clots at a higher rate vis-à-vis men. As we know, women’s estrogen strengthens their immunity; hence, the use of hormonal contraceptive pills can increase their immune response and make them more sensitive. It is also undeniable that a person’s physical health status can be deemed to be causing VITT.

## 13 VITT diagnosis

At first glance, the VITT recognition seems comparatively straightforward. However, numerous immune thrombocytopenia cases have been reported following the injection of Moderna and Pfizer vaccines. It is worth noting that other clinical conditions also can raise anti-PF4 antibodies in the blood (e.g., after cardiovascular surgery, and even in healthy people although VITT is not ruled out) [48].

The evidence indicates that venous or arterial thrombosis might occur in uncommon sites such as the brain or stomach in specific individuals, with clinical signs appearing 5–20 days after immunization. If followed by thrombocytopenia, this event can lead to an adverse impact of the preceding COVID-19 vaccination [46]. Because the mortality rate for individuals with cerebral venous thrombosis is more significant than predicted, early treatment actions are considered crucial [49].

## 14 Differences and causes of the effects of various vaccines on blood clots

In the following section, the vaccines whose injection has been officially reported to be accompanied by VITT and blood clots in the receivers will be discussed.

### 14.1 AstraZeneca COVID-19 vaccine

ChAdOx1 nCoV-19 (also named Vaxzevria) is a low-cost and easy-to-store vaccine manufactured by the Oxford University and AstraZeneca. It also has been the most controversial COVID-19 vaccine due to the diverse reports of thrombosis following its administration [50]. The thrombotic events after Vaxzevria vaccination was reported in many countries including Germany, Austria, and Norway [46,51]. It has been stated that patients diagnosed with TTS have developed one or more thrombotic events such as cerebral venous sinus thrombosis (CVST), splanchnic vein thrombosis, pulmonary embolism, or deep vein thrombosis (DVT). However, CVST was the most prevalent one, which mainly occurred before secondary cerebral hemorrhage [50]. The laboratory tests in VITT patients indicated low fibrinogen and platelet counts but dramatically high D-dimer concentration [52]. As mentioned earlier, the thrombotic events in such patients were caused by the synthesis of antibodies against platelet factor 4 (PF4) and incommensurate activation of the immune system.

### 14.2 Pfizer/Biontech COVID-19 vaccine

BNT162b1 (known as Comirnaty) produced by BioNTech and Pfizer is the first authorized COVID-19 vaccine and the first vaccine utilizing mRNA technology in the vaccine history [53]. This vaccine is reported to have the highest efficacy in preventing symptomatic COVID-19, but it is also the most expensive and hard-to-restore vaccine. In fact, it needs to be stored at −60°C [50,54]. Thrombotic events have been officially reported after the Pfizer COVID-19 vaccine. DVT was confirmed in one case in February 2021 [55]. Moreover, the first case on immune thrombocytopenia was also reported in a 22-year-old patient suffering from gum bleeding and petechiae after vaccination with Comirnaty in January 2021 [56]. Although these events are closely related to blood, they cannot accurately be counted as VITT or TTS; therefore, such cases can be purely coincidental despite the incidence rate (reported to be 3.3 per 100,000) [57]. However, it could be obviously concluded that VITT is presumably caused by the production of antibodies against PF-4 in patients who demonstrate TTS symptoms after vaccination [50].

### 14.3 Moderna COVID-19 vaccine

mRNA-1273 or Moderna COVID-19 vaccine is one of the other mRNA-based vaccines, which is produced by Moderna company [58]. A study conducted by Gee et al. reported that almost all (i.e., 90.8%) of the side effects after vaccination with mRNA-1273 could be categorized as nonserious adverse effects [59]. The first related event to clotting was reported in a 60-year-old man who experienced thrombocytopenia (but not a VITT/TTS) and purpuric after being vaccinated by Moderna COVID-19 vaccine [60]. However, the incidence of acute deep vein thrombosis (ADVT) was also reported as a blood clotting-related event after Moderna vaccination [61]. Thrombosis after Moderna vaccination is probably related to anti-PF4 antibodies such as previously mentioned vaccines.

### 14.4 Janssen’s COVID-19 vaccine

Ad26.COV2 (also branded as J&J COVID-19 vaccine), which was approved by the United States Food and Drug Administration (US-FDA) in February 2021, is one of the most controversial COVID-19 vaccines. According to various reports, rare and unknown types of thrombosis have been witnessed [62,63]. Similar to VITT in patients vaccinated by the AstraZeneca COVID-19 vaccine, CSVT and splanchnic veins thrombosis were also reported in individuals immunized by the J&J vaccine [50], corroborating the role of anti-PF4 synthesis in VITT. The causes and effects of VITT in the aforementioned vaccines were primarily similar, but it is worthy of noting prescribing anti-Xa inhibitors first-line anticoagulants. Avoiding the use of heparin is strongly advised for VITT patients who had received viral vector-based COVID-19 vaccine [64].

### 14.5 Other COVID-19 vaccines

A study by Liu et al. [65] targeted 406 healthcare employees who had received BBIBP-CorV Sinopharm vaccine (as a most common inactivated-virus-based vaccine) to evaluate the occurrence of VITT or other thrombotic-related events by measuring ten autoantibodies before and after vaccination [65]. The elicited results testify that although the concentration of anti-PF4-heparin antibodies has elevated in seven cases, none of the 406 cases showed VITT or thrombotic-related disorders [65], which evidently indicates more safety in the inactivated virus-based vaccines at VITT. It can also be mentioned that no official VITT cases were reported for Sputnik V as a viral vector-based vaccine. Argentina and Serbia – as two widely consumers of Sputnik V vaccine where a large number of this vaccine was inoculated – reported no blood clotting or thrombotic-related events [66,67]. However, it is clear that the causes and effects of different vaccines, which cause VITT on blood clots, are almost the same and no significant difference can be perceived among them.

## 15 Clinicians should consider these recommendations when facing patients with VITT

Anti-PF4-heparin and PF4-polyanion antibodies can be tested alone or together using a commercially approved method. However, false-negative results are possible in some rapid immunoassays and chemo-luminescence tests. ISTH guidelines recommend affirmation employing a functional test such as carbon 14-labeled serotonin discharge measure. The preliminary assessment of suspected VITT ought to incorporate a total blood cell tally, D-dimer and fibrinogen levels, PF4-polyanion ELISA, and imaging [48]. Overall, laboratory assessment of all important blood factors is helpful for VITT diagnosis and treatment. However, D-dimer concentration and detection of thrombus incidence by imaging methods play a more essential and critical role in proposed algorithms for VITT management [68].

Alternative causes of thrombocytopenia and thrombosis must be considered and examined regardless of (autoimmune) HIT and VIPIT test results. Several thrombotic microangiopathy syndromes such as antiphospholipid syndrome, immunological thrombotic-thrombocytopenic purpura or atypical hemolytic uremic syndrome, paroxysmal nocturnal hemoglobinuria, and underlying malignant (hematological) illnesses are only a few examples that can be counted [37].

## 16 VITT treatment

VITT treatment must be based on clinical and laboratory analyses. That said, this can be modulated depending on the case condition. Intravenous immunoglobulin, glucocorticoids, and anticoagulation are significant parts of VITT therapy. Platelet-mediated activation of the Fcγ receptor is inhibited by high-dose intravenous immunoglobulin [48]. The research guideline is in line with previous findings in the management of severe A-HIT. A high dose of intravenous immune globulin has led to quickly rising platelet and hypercoagulability de-escalation. The addition of immunoglobulin was beneficial in suppressing platelet activation by patients’ antibodies [46]. It should be noted that HIT/VIPIT diagnostics should be requested before IVIG is given since high-dose immunoglobulins might cause false-negative test results [37].

Anticoagulant alternatives must include nonheparin anticoagulants that are utilized for heparin-induced thrombocytopenia therapy. A compelling experiment has ruled out the heparin-dependent increase of platelet activation [46]. Although heparin does not present worse results with certainty, it is better not to use it for VITT patients. Heparin should not be prescribed to VITT patients although it has not been conclusively proven to have harmful consequences. Anticoagulants except for heparin are preferable (e.g., Argatroban and direct oral anticoagulants). In several countries, the USA’s Danaparoid has been prescribed. Platelets should not be transfused since they increase platelets to activate and provide antibody-mediated blood clotting [49]. Platelet transfusions may aggravate the thrombotic signs of VITT and should be avoided unless life-threatening bleeding is occurring [48].

## 17 World Health Organization’s latest recommendations for VITT and TTS treatment following vaccination

World Health Organization has constantly observed the TTS occurrence after COVID-19 vaccination and provides some recommendations, advises, and caution for VITT patients in its published guideline named “Guidance for clinical case management of thrombosis with thrombocytopenia syndrome (TTS) following vaccination to prevent coronavirus disease (COVID-19)” [69] based on the official reports. The most significant WHO recommendations for VITT patients advise against the use of heparin, infusion of platelet, and steroid treatment in TTS patients [69]. WHO also stipulates that a platelet infusion strategy should be adopted for TTS patients who suffer from severe thrombocytopenia (platelets < 50,000/µL) or who are in an emergency situation. It should also be noted that WHO explicitly advises the injection of intravenous immunoglobulins (IVIG) and/or nonheparin-based anticoagulants for the vaccine receivers who have been diagnosed with TTS/VITT [69].

WHO also recommends that for preventing exposure to the antigen, patients who have reported VITT should not receive the same vaccine as the booster dose. It also strongly advises against the use of heparin-based drugs and vaccination with adenovirus vector-based vaccines in individuals with a history of HIT [69]. Recently, different successful treatments of definite VITT were reported [68], in which nonheparin anticoagulation, along with IVIG, and prednisone were prescribed as the primary therapeutic agents [70].

## 18 Blood clots statistics

Although there is a connection between some COVID-19 vaccines and blood clots, it is not suggested to break on vaccination. Researchers at a new pre-print study from Oxford University have demonstrated that the rate of CVST AstraZeneca vaccination compared with the rate of CVST associated with COVID-19 is 8–10. This research was based on a U.S. health database and provided cogent evidence demonstrating the benefits of vaccines over and above its risks. All crucial information on COVID-19 vaccines is summarized in Table 1.

**Table 1:** The most crucial information on mentioned COVID-19 vaccines in this article

Vaccine’s scientific name	Used technology	Made by	Common side effects	Rare side effects	Thrombosis reported after injection	Efficacy^A^	Refs.
BNT162b2	mRNA-based	Pfizer–BioNTech companies	Allergy	Myocarditis	Yes	94%	[71–76]
CX-024414	mRNA-based	Moderna company	Pain, fatigue, headache, myalgia, and arthralgia	Myocarditis and pericarditis	Yes	66–98%	[36,76–79]
ChAdOx1 nCoV-19	Modified adenovirus vector based	Oxford University and AstraZeneca company	Vomiting, diarrhea, fever, and swelling	Thrombosis with thrombocytopenia syndrome, and anaphylaxis	Yes	92%	[52,80–83]
Sputnik V	Modified adenovirus vector based	Gamaleya Research Institute of Epidemiology and Microbiology	Mild adverse effects	—	Not reported	68–88%	[66,84–87]
Ad26.COV2.S	Modified adenovirus vector based	Johnson & Johnson company	Pain, headache, tiredness, muscle pain, and nausea	Thrombosis with thrombocytopenia syndrome and anaphylaxis	Yes	71%	[63,88–91]
NVX-CoV2373	Protein subunit	Novavax and the Coalition for Epidemic Preparedness Innovations	Headache, nausea (feeling sick) or vomiting, muscle and joint pain, and tenderness	Anaphylaxis	Not reported	100% (alpha variant)	[92–94]
Sinopharm BIBP COVID-19 vaccine	Inactivated virus	Sinopharm Institute of Biological Products	Injection site pain and fatigue	—	Not reported	67–70%	[95–98]
Covaxin BBV152	Inactivated virus	Bharat Biotech with the Indian Council of Medical Research - National Institute of Virology	Injection site pain, Head ache, fatigue, myalgia, malaise, and pyrexia	—	Not reported	33–62%	[99–101]

^A^The effectiveness of all mentioned vaccine means their efficiency for preventing hospitalization and is measured against Delta variant.

## 19 Conclusion

Adenovirus vector vaccines (AstraZeneca, Janssen, Sputnik V [no official case reported]) seem to be responsible for developing vaccine-induced immune thrombotic thrombocytopenia. However, it is not entirely understood what exactly triggers the immune system to cause VITT. That said, there are some speculations about DNA/RNA-PF4 complex, Spike protein itself, and transcription complexities that describe the occurrence of thrombotic thrombocytopenia syndrome reasonably. In addition, some factors like the patient’s underlying disease or age/sex and hormonal situation of the patient are effective in the management and treatment of VITT. All in all, it appears that first and foremost, physicians should review the testes’ results of anti-PF4-heparin and PF4-polyanion antibodies. Assessment of D-dimer and fibrinogen levels, as well as imaging methods, may also be helpful for medical doctors in evaluating suspected VITT patients. WHO has recommended the use of IVIG and/or nonheparin-based anticoagulants in individuals who are suffering from TTS after the COVID-19 vaccination. It has also generally (not in exceptional cases) advised against the use of heparin, platelet infusion, and steroid treatment in such patients. It should also be noted that although there is a connection between blood clots and COVID-19 vaccination, statistics and data have provided cogent proof that the occurrence of blood clots in COVID-19 is up to 10 times more common than the vaccines’ injection.
